# Electrocardiographic Predictors of Coronary Heart Disease and Sudden Cardiac Deaths in Men and Women Free From Cardiovascular Disease in the Atherosclerosis Risk in Communities Study

**DOI:** 10.1161/JAHA.113.000061

**Published:** 2013-06-21

**Authors:** Pentti M. Rautaharju, Zhu‐Ming Zhang, James Warren, Richard E. Gregg, Wesley K. Haisty, Anna M. Kucharska‐Newton, Wayne D. Rosamond, Elsayed Z. Soliman

**Affiliations:** 1 Epidemiological Cardiology Research Center (EPICARE), Division of Public Health Sciences, Wake Forest School of Medicine, Winston‐Salem, NC (P.M.R., Z.M.Z., E.Z.S.); 2 Dalhousie University Medical School, Halifax, Nova Scotia, Canada (J.W.); 3 Advanced Algorithms Research Center, Philips Healthcare, Thousand Oaks, CA (R.E.G.); 4 Department of Medicine, Section on Cardiology, Wake Forest School of Medicine, Winston‐Salem, NC (W.K.H., E.Z.S.); 5 Department of Epidemiology and Community Health, Gillings School of Global Public Health, University of North Carolina at Chapel Hill, Chapel Hill, NC (A.M.K.N., W.D.R.)

**Keywords:** electrocardiography, ischemic heart disease, prognosis, repolarization, sudden death

## Abstract

**Background:**

We evaluated predictors of coronary heart disease (CHD) death and sudden cardiac death (SCD) in the Atherosclerosis Risk in Communities (ARIC) study.

**Methods and Results:**

The study population included 13 621 men and women 45 to 65 years of age free from manifest cardiovascular disease at entry. Hazard ratios from Cox regression with 95% confidence intervals were computed for 18 dichotomized repolarization‐related ECG variables. The average follow‐up was 14 years. Independent predictors of CHD death in men were TaVR‐ and rate‐adjusted QTend (QT_ea_), with a 2‐fold increased risk for both, and spatial angles between mean QRS and T vectors and between Tpeak (T_p_) and normal R reference vectors [θ(R_m_|T_m_) and θ(T_p_|T_ref_), respectively], with a >1.5‐fold increased risk for both. In women, independent predictors of the risk of CHD death were θ(R_m_|T_m_), with a 2‐fold increased risk for θ(R_m_|T_m_), and θ(T_p_|T_ref_), with a 1.7‐fold increased risk. Independent predictors of SCD in men were θ(T_p_|T_ref_) and QT_ea_, with a 2‐fold increased risk, and θ(T_init_|T_term_), with a 1.6‐fold increased risk. In women, θ(T_init_|T_term_) was an independent predictor of SCD, with a >3‐fold increased risk, and θ(R_m_|T_m_) and TV1 were >2‐fold for both.

**Conclusions:**

θ(R_m_|T_m_) and θ(T_p_|T_ref_), reflecting different aspects of ventricular repolarization, were independent predictors of CHD death and SCD, and TaVR and TV1 were also independent predictors. The risk levels for independent predictors for both CHD death and SCD were stronger in women than in men, and QT_ea_ was a significant predictor in men but not in women.

## Introduction

Evaluation of the risk of adverse cardiac effects for QT prolongation has been the focal point of numerous clinical and epidemiological studies.^1^ However, QT is known to have notable limitations,^2–4^ and professional organizations and governmental regulatory agencies have recognized the need for more sensitive predictors of adverse cardiac events than QT.^5–7^ There is limited information about the utility of repolarization subintervals and associated repolarization‐related ECG variables for prediction of adverse cardiac events. However, several investigations have found increased mortality risk in various clinical and general populations for the spatial angle between the mean QRS and T vectors [θ(R_m_T_m_)].^8–12^

The objective of our study was to evaluate the risk of coronary heart disease (CHD) death and sudden cardiac death (SCD) in cardiovascular disease (CVD)–free men and women for a comprehensive set of repolarization‐related ECG parameters.

## Methods

### Study Population

The Atherosclerosis Risk in Communities (ARIC) study was designed as a prospective investigation of the cause and natural history of atherosclerosis, its clinical manifestations, and the community burden of CHD. Risk factors were measured and outcomes evaluated in a population‐based probability sample of adults 45 to 65 years of age at the 1987 to 1989 baseline examination; follow‐up of the cohort is ongoing. Study population and definitions of prevalent diseases at baseline and outcomes have been described previously.^13–15^ Deaths were classified into definite or possible CHD death, non‐CHD death, and unclassified death.

SCD was defined as definite or possible CHD death that occurred within 1 hour after the onset of acute symptoms. CHD at baseline was classified by angina pectoris using the questionnaire of Rose et al^16^ or myocardial infarct (MI), defined by a self‐reported episode requiring hospitalization for >1 week, MI diagnosed by a physician, major Q waves at the baseline ECG (Minnesota Code 1.1),^17^ or previous coronary artery bypass graft or coronary angioplasty. Prevalent (baseline) heart failure (HF) was determined on the basis of evidence of use of HF‐related medications and classified according to Gothenburg criteria.^18^ Baseline cerebrovascular disease was defined as self‐reported stroke or transient ischemic attack that was verified by a study physician's review of the reported symptoms.

After exclusion of ECGs of participants with bundle branch blocks, artificial pacemakers, Wolf‐Parkinson‐White pattern, and technical errors in ECG recording detected in visual inspection of all the study ECGs using computer graphics terminals, source data for the present investigation were available from 15 005 ARIC participants. Participants with CVD at baseline (n=1384) were excluded from the present study (CHD, hospitalized HF, or cerebrovascular disease classified by criteria as noted above), leaving a CVD‐free subgroup of 5937 men and 7684 women for the present study. The outcome data for CHD and SCD were available from a mean follow‐up period of 14 years.

### Electrocardiographic Procedures and Quality Control

Standardized procedures were used for recording the 12‐lead ECGs with MAC Personal Computer (Marquette Electronics, Milwaukee, WI) in each clinical center. ECGs were processed in a central ECG laboratory initially using the Dalhousie Novacode ECG program.^19^ All ECGs were later reprocessed with the GE Marquette 12‐SL program (GE Marquette, Milwaukee, WI). The quasi‐orthogonal XYZ leads were derived from the 8 linearly independent component‐leads of the 12‐lead ECG signals using Kors' transformation,^20^ and these leads were used as the source data to derive ECG parameters for the repolarization model.

QTpeak (QT_p_), QTend (QT_e_), and QTonset (QT_o_) intervals were first rate‐adjusted as power functions of the RR interval derived in CVD‐free men and women by regressing lnQT on lnRR. The exponent for RR was 0.33 for QT_e_ for men and women and ranged from 0.36 to 0.40 for QT_p_ and QT_o_. It was noticed that as long as proper functional form was used for rate adjustment, the exact value of the exponent for the RR interval had little influence on the R‐square values. Additional analyses were performed to compare the above exponential rate adjustment formulas with simpler linear functions of the RR interval in the CVD‐free groups of men and women (Table 1). The results revealed that the differences in the accuracy of rate adjustment in terms of the R‐square values were quite small. Recognizing that using different rate adjustment functions for QT_e_ and QT_p_ is an added complexity, a simpler QT_p_ rate adjustment as the difference (QT_ea_–T_p_T_e_ interval) was derived as shown in the middle section of Table 1. This became possible because the T_p_T_e_ interval in the CVD‐free groups was practically independent from heart rate (R‐square, 0.120 for men and 0.045 for women).

**Table 1.** Rate‐Adjustment Formulas for QTend, QTpeak, and QTonset by Linear (Top Section) and Power (Bottom Section) Functions of the RR Interval by Sex

	Adjustment Formulas	R‐Square
Linear functions		
QT_e_*		
Men	QT_ea_=QT_e_+127×(1−RR)	0.571
Women	QT_ea_=QT_e_+136×(1−RR)	0.529
QT_p_*		
Men	QT_pa_=QT_p_+116×(1−RR)	0.496
Women	QT_pa_=QT_p_+130×(1−RR)	0.477
QT_o_*		
Men	QT_oa_=QT_o_+84×(1−RR)	0.451
Women	QT_oa_=QT_o_+100×(1−RR)	0.422
QT_p_		
Men and women	QT_pa_=QT_ea_−T_p_T_e_*	
Exponential functions		
QT_e_		
Men	QT_ea_=QT_e_+416×(1−RR^1/3^)	0.571
Women	QT_ea_=QT_e_+435×(1−RR^1/3^)	0.529
QT_p_		
Men	QT_pa_=QT_p_+295×(1−RR^0.40^)	0.498
Women	QT_pa_=QT_p_+303×(1−RR^0.40^)	0.436
QT_o_		
Men	QT_oa_=QT_o_+206×(1−RR^0.40^)	0.452
Women	QT_oa_=QT_o_+238×(1−RR^0.40^)	0.425

RR interval is in seconds, other intervals in milliseconds.

* QT_ea,_ QT_pa_, and QT_oa_ refer to rate‐adjusted QTend (QT_e_), QTpeak (QT_p_), and QTonset (QT_o_), respectively.

* An alternative QT_pa_ formula as (QT_ea_−T_p_T_e_ interval) with linear QT_ea_ of men and women on top of the table.

### Definitions of Repolarization Parameters

A set of 18 repolarization‐related ECG variables was chosen for evaluation based on previous data of the variables' value as risk predictors or because of their functional role in the generation of normal and abnormal repolarization waveforms. QRS duration was included among these repolarization‐related parameters because even moderate QRS prolongation has been shown to induce secondary repolarization abnormalities associated with adverse cardiac events.

**Figure 1.**
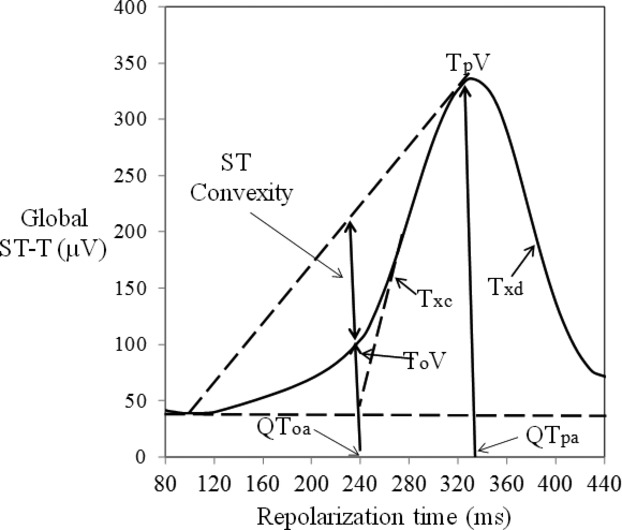
ST‐T vector magnitude curve with temporal landmarks and measurement points for key intervals and amplitudes in the repolarization model. The peak of the global T wave (T_p_) coincides with the rate‐adjusted QTpeak (QT_pa_). The time point of Tonset (T_o_) corresponding to the rate‐adjusted QTonset interval (QT_oa_) is obtained by extrapolating the line from the time of maximum slope at T‐wave upstroke (T_xc_) to the intersection with the horizontal line drawn from the minimum ST after the J‐point. The time of the minimum slope at the T‐wave downstroke (T_xd_) defines the end point of the T_p_T_xd_ interval conceived in the repolarization model to represent initial left ventricular repolarization time (RT) dispersion. Double arrow signifies ST segment convexity as the difference of the ST segment from the gradient line from the ST onset to T_p_.

The conceptual model used to derive the repolarization parameters for the present study has been described in previous publications.^21–22^ Temporal landmarks and measurement points for key intervals and amplitudes in the repolarization model are shown in the sketch of the ST‐T vector magnitude curve in Figure 1. The time of T onset (T_o_) corresponding to the rate‐adjusted QTonset interval (QT_oa_) in reference to QRS onset was obtained by extrapolating the line from the point of maximum slope at T‐wave upstroke (T_xc_) to the intersection with the horizontal line drawn from the minimum ST after the J‐point. Time of the minimum slope at T‐wave downstroke (T_xd_) defines the end point of the T_p_T_xd_ interval, which is conceived in the repolarization model to represent initial left ventricular repolarization time (RT) dispersion. RT peak (RT_p_) is computed as a function of the rate‐adjusted QTpeak interval (QT_pa_). Briefly, RT_p_=QT_pa_−{[Cos θ(T_p_|T_ref_)−1]×[T_p_T_xd_]}/2, where θ(T_p_|T_ref_) is the spatial angle between T_p_ vector and T_ref_ is the reference mean T vector direction in normal repolarization (sex‐ and race‐specific components of the unit vectors of T_ref_ are listed in the footnote of Table 3). T_p_–T_xd_, in turn, is the interval from T_p_ to T_xd_, where T_xd_ is the inflexion point (the minimum slope) at global T‐wave downstroke. Left ventricular (LV) RT at point T_xd_ (RT_xd_) is obtained with an algorithm similar to that for RT_p_, whereby RT_xd_=QT_pa_+{[Cos θ(T_p_|T_ref_)+1]×[T_p_T_xd_]}/2. The key role of RT_p_ and RT_xd_ for deriving ECG estimates for RT_epi_ and RT_endo_ is considered in detail in the Discussion section in the subsection “Validity of the Repolarization Model.”

In addition to θ(T_p_|T_ref_), a number of other spatial angles reflecting deviations of the direction of repolarization from the reference normal direction during various repolarization subintervals and other repolarization‐related interval and amplitude variables were used in various phases of the study. Their definitions are listed in the footnotes of the corresponding tables.

### Statistical Methods

One baseline ECG per participant was used for all analyses. Mean values and standard deviations were determined for continuous variables and frequencies and percents for categorical variables. Cox proportional hazards regression was used to assess associations of ECG variables with the risk of CHD death and SCD using both univariable and multivariable risk models. Predictor ECG parameters were first evaluated as continuous variables and then dichotomized using quintiles to define test and reference groups. The thresholds for test groups are listed in Table 3. Hazard ratios (HRs) were evaluated for increased values of the ECG parameters (quintile 5) as the test group, with quintiles 1 to 4 as the reference group. However, quintile 1 corresponding to decreased values was used as the test group for TaVL and T_p_V, with the remaining 4 quintiles as the reference group. ECG predictors were first evaluated as unadjusted single variables and subsequently in multivariable‐adjusted models with adjustment for age, sex, center, race, education, smoking status, diabetes, hypertension, family history of CHD/stroke, BMI, SBP, ratio of total cholesterol/high‐density lipoprotein, glucose, creatinine, and uric acid. An association was considered significant when *P*<0.05 and no adjustment for multiplicity of comparisons had been considered. Finally, to identify independent ECG risk predictors, those ECG variables that were significant predictors in single variable models were entered simultaneously into the Cox regression model, and each was adjusted to other ECG variables.

Participants who had no events during the follow‐up period were censored in the analysis at their date of last contact. A participant who died from CHD with the death not SCD was censored at the date of the CHD non‐SCD death. No attempt was made to evaluate possible competing risks. All analyses were performed with SAS version 9.1.3 (SAS Institute, Cary, NC).

## Results

### Study Group Characteristics

Demographic and clinical characteristics of the study population listed in Table 2 have been described in more detail in previous publications.^21,23^ The age range of the study population was 45 to 65 years, with mean age of 54 years (SD, 5.7 years). The study population was predominantly white (73%). The prevalence of hypertension was ≈30% in men and women.

**Table 2.** Key Demographic/Clinical Characteristics of Study Population by Sex (Mean [SD] or Number [%])

	Men	Women	*P* Value
Age, y	54.2 (5.8)	53.6 (5.7)	<0.001
Body mass index, kg/m^2^	27 (4.1)	28 (6.0)	0.012
White, n (%)	4563 (76.9)	5450 (70.9)	<0.001
Current smokers, n (%)	1623 (27.4)	1877 (24.5)	<0.001
Systolic blood pressure, mm Hg	122 (17.5)	120 (19.2)	<0.001
Hypertensives, n (%)	1837 (31.1)	2442 (31.9)	0.288
Diabetes mellitus, n (%)	621 (10.5)	785 (10.3)	0.681
MI by MC criteria, %	3.0	1.4	<0.001
LVH by Cornell voltage, %	1.5	3.9	<0.001

MI indicates myocardial infarction; MC, Minnesota Code; LVH, left ventricular hypertrophy.

ECG variables including all repolarization‐related parameters evaluated are listed in Table 3. Most of the differences between men and women were statistically significant. QT_pa_, the rate‐adjusted QTpeak, was 18 ms shorter and QT_oa_, the rate‐adjusted QTonset, 21 ms shorter in men than in women. The sex difference in global rate‐adjusted QT (QT_ea_) was smaller, 10 ms. θ(R_m_|T_m_), the spatial angle between the mean QRS and T vectors, was 12° wider in men than in women. Among other notable differences in ECG parameters, T_o_V, Tonset vector magnitude, was ≈30% lower in women than in men, and a similar difference was observed in T_p_V, the spatial magnitude of the Tpeak vector.

**Table 3.** Means (SDs) for Electrocardiographic Variables by Sex and Threshold Values for Test Quintiles Used for Risk Evaluation Models in Tables 3 through 7

	Mean (SD)		*P* Value*	Test Group Threshold	
	Men	Women		Men	Women
Heart rate/min	65 (10.2)	67 (10.0)	<0.001	>73	>75
QRS duration, ms	95 (9.1)	87 (8.3)	<0.001	102	96
RNDPV,* μV	54 (22.6)	43 (17.4)	<0.001	69	56
QT_ea_,* ms	413 (16.4)	423 (17.5)	<0.001	426	437
QT_oa_,* ms	216 (18.3)	237 (15.6)	<0.001	199	222
QT_pa_,* ms	311 (18.1)	329 (19.8)	<0.001	268	207
RT_epi_,* ms	316 (17.8)	332 (18.8)	<0.001	329	347
RT_endo_,* ms	348 (19.6)	366 (20.1)	<0.001	364	381
T_p_T_xd_,* ms	36 (14.7)	38 (15.3)	<0.001	48	50
θ(T_p_T_ea_)a,* ms	100 (13.9)	95 (15.7)	<0.001	110	106
θ(R_m_|T_m_),* (°)	58 (26.8)	4 (24.4)	<0.001	71	56
θ(T_p_|R_ref_),* (°)	21 (15.7)	21 (19.0)	0.0151	28	30
θ(T_init_|T_term_),* (°)	18 (11.6)	16 (11.1)	<0.001	24	23
T complexity*	0.34 (0.16)	0.36 (0.18)	<0.001	0.47	0.51
TaVR, μV	−219 (96.9)	−201 (86.7)	<0.001	−146	−131
TaVL, μV	94 (95.6)	75 (80.3)	<0.001	24	0
TV1, μV	−133 (146.0)	−12 (119.6)	<0.001	244	122
ST_o_V,* μV	54 (27.9)	36 (19.7)	<0.001	75	53
T_o_V, μV	148 (56.7)	104 (41.0)	<0.001	194	138
T_p_V, μV	418 (157)	336 (132)	<0.001	268	207
VT_o_/VT_p_,* μV	0.39 (0.09)	0.36 (0.11)	<0.001	0.45	0.42

HRs were evaluated for quintile 5 (quintile 1 for QT_oa_, TaVL, and T_p_V) as the test group, with the remaining 4 quintiles as the reference group. SD indicates standard deviation; HR, hazard ratio; CI, confidence interval.

* *P* values for *z* test for ratios and for *t* test for sex differences.

* RNDPV=QRS nondipolar voltage from singular value decomposition (square root of pooled variance of components 4 to 8).

* QT_ea_, QT_pa_, and QT_oa_ refer to rate‐adjusted QTend (QT_e_), QTpeak (QT_p_), and QTonset (QT_o_) intervals, respectively, using linear formulas listed in Table 1.

* RT_epi_ and RT_endo_ denote subepicardial and subendocardial repolarization times (see Methods and Results section).

* T_p_T_xd_=T_p_T_xd_ interval representing dispersion of the initial left ventricular repolarization.

* θ(T_p_T_ea_)a, global repolarization time dispersion (interval from QT_pa_ to QT_ea_).

* θ(R_m_|T_m_), spatial angle between the mean QRS and T vectors.

* θ(T_p_|T_ref_), spatial angle between T_p_ vector and the T reference (T_ref_) vector.

* θ(T_init_|T_term_), spatial angle between initial and terminal T vectors.

* T‐wave complexity, ratio of the second to the first principal component from singular value decomposition of the T wave.

* Symbol “V” in ST_o_V, T_o_V, and T_p_V refers to spatial magnitudes of ST_o_, T_o_, and Tp vectors, respectively.

* T_o_V/T_p_V is the ratio of the T_o_ and T_p_ spatial vector magnitudes.

### CHD Death Predictors

Summary results are presented Table 4 for ECG variables evaluated in Cox regression as unadjusted single predictors and as multivariable adjusted single ECG predictors. From the set of 18 ECG variables, 12 in men and 13 in women were significant predictors of CHD death in unadjusted single ECG variable models. The set of significant unadjusted single predictors was the same in men and in women except that QRS duration was a significant predictor in women but not in men. Angular variables and T‐wave amplitudes were the strongest single predictors of CHD mortality risk. Seven of the predictors in men and 5 in women remained significant after adjustment for demographic and clinical risk factors.

**Table 4.** Hazard Ratios With 95% Confidence Intervals for ECG Predictors of Coronary Heart Disease Death in Men and Women

	Men				Women			
	Univariable*	*P* Value	Multivariable*	*P* Value	Univariable	*P* Value	Multivariable	*P* Value
QRS duration, ms	1.26 (0.93 to 1.72)	0.143	1.33 (0.97 to 1.82)	0.081	1.57 (1.09 to 2.25)	0.015	1.19 (0.81 to 1.76)	0.372
RNDPV*	1.30 (0.95 to 1.78)	0.099	1.21 (0.85 to 1.71)	0.297	1.24 (0.83 to 1.86)	0.230	1.03 (0.66 to 1.60)	0.372
QT_ea_,* ms	1.88*** (1.42 to 2.50)	<0.001	1.49 (1.11 to 2.00)	0.084	1.74 (1.21 to 2.50)	0.003	1.25 (0.85 to 1.85)	0.365
T_p_T_xd_,* ms	1.11 (0.81 to 1.52)	0.508	0.94 (0.68 to 1.30)	0.720	0.86 (0.56 to 1.33)	0.499	0.65 (0.40 to 1.06)	0.084
θ(T_p_T_e_)a,* ms	1.35 (0.99 to 1.82)	0.051	1.14 (0.83 to 1.57)	0.416	1.41 (0.96 to 2.06)	0.080	0.92 (0.60 to 1.41)	0.695
RT_epi_,* ms	1.77 (1.33 to 2.34)	<0.001	1.46 (1.08 to 1.97)	0.014	1.90 (1.32 to 2.73)	<0.001	1.33 (0.90 to 1.98)	0.153
θ(R_m_|T_m_),* (°)	2.12 (1.61 to 2.79)	<0.001	1.46 (1.09 to 1.95)	0.012	3.85 (2.75 to 5.40)	<0.001	2.32 (1.59 to 3.39)	<0.001
θ(T_p_|T_ref_),* (°)	2.46 (1.88 to 3.23)	<0.001	1.85 (1.39 to 2.46)	<0.001	3.31 (2.35 to 4.64)	<0.001	1.61 (1.09 to 2.36)	0.016
θ(T_init_|T_term_),* (°)	2.12 (1.61 to 2.79)	<0.001	1.58 (1.18 to 2.11)	0.002	2.92 (2.07 to 4.11)	<0.001	1.81 (1.23 to 2.66)	0.003
T complexity*	1.07 (0.78 to 1.47)	0.661	0.63 (0.69 to 1.32)	0.783	1.23 (0.83 to 1.81)	0.302	1.01 (0.67 to 1.53)	0.970
TaVR amplitude, μV	2.56 (1.96 to 3.34)	<0.001	1.66 (1.25 to 2.22)	<0.001	2.94 (2.09 to 4.13)	<0.001	1.54 (1.05 to 2.25)	0.027
TV1 amplitude, μV	1.57 (1.17 to 2.11)	0.003	1.22 (0.89 to 1.66)	0.224	3.18 (2.26 to 4.46)	<0.001	1.89 (1.29 to 2.76)	0.001
ST_o_V,* μV	1.38 (1.01 to 1.89)	0.045	1.13 (0.79 to 1.60)	0.502	1.87 (1.29 to 2.71)		1.04 (0.67 to 1.62)	0.851
TVo, μV	1.25 (0.91 to 1.73)	0.171	1.22 (0.85 to 1.76)	0.273	1.14 (0.76 to 1.74)	0.525	0.82 (0.51 to 1.33)	0.425
T_p_V, μV	0.82 (0.57 to 1.17)	0.271	1.02 (0.85 to 1.56)		0.74 (0.46 to 1.19)	0.212	0.66 (0.59 to 1.65)	0.967
T_o_V/T_p_V*	1.79 (1.34 to 2.40)		1.11 (0.69 to 1.50)	0.936	2.10 (1.47 to 3.00)	<0.001	0.98 (0.64 to 1.50)	0.919

HR was evaluated for increased values of the ECG parameters (quintile 5) as the test group, with quintiles 1 to 4 as the reference group. However, quintile 1 corresponding to decreased values was used as the test group for TaVL and T_p_V, with the remaining 4 quintiles as the reference group. HR indicates hazard ratio; CI, confidence interval; CHD, coronary heart disease; HDL, high‐density lipoprotein; LDL, low‐density lipoprotein.

* Univariable refers to unadjusted single ECG variable model and multivariable to single ECG variable multivariable‐adjusted risk model adjusted for age, race, education level, smoking status, alcohol status, asthma, cancer, diabetes, hypertension, family history of CHD and stroke, body mass index, systolic and diastolic blood pressure, HDL, LDL, triglycerides, white blood count, glucose, creatinine, and uric acid. HRs were evaluated for quintile 5 (quintile 1 for QT_oa_,T_amp_.aVL and T_p_V) as the test group, with the remaining 4 quintiles as the reference group.

* RNDPV, QRS nondipolar voltage from singular value decomposition (square root of pooled variance of components 4 to 8).

* QT_ea_, rate‐adjusted QRe where QT_ea_=QT_e_+127 (1−RR) for men and QT_ea_=QT_e_+136 (1−RR) for women.

* T_p_T_xd_=T_p_T_xd_ interval representing dispersion of the initial left ventricular repolarization.

* θ(T_p_T_e_)a, global repolarization time dispersion (interval from QT_pa_ to QT_ea_).

* RT_epi_ denotes subepicardial repolarization time (see Methods and Results section).

* θ(R_m_|T_m_), spatial angle between mean QRS and T vectors.

* θ(T_p_|T_ref_), spatial angle between T_p_ vector and the T reference (T_ref_) vector.

* θ(T_init_|T_term_), spatial angle between the initial T vectors from quintiles 1 to 3 and the terminal T vectors from quintiles 4 to 5.

* T‐wave complexity, ratio of the second to the first principal component from singular value decomposition of the T wave.

* Symbol “V” in ST_o_V, T_o_V, and T_p_V refers to the spatial magnitudes of the ST_o_, T_o_, and Tp vectors, respectively.

* T_o_V/T_p_V is the ratio of the T_o_ and T_p_ spatial vector magnitudes.

### Sudden Cardiac Death Predictors

As for CHD death, many repolarization‐related variables (11 in men and 11 in women) were significant predictors of SCD when evaluated as unadjusted single ECG‐variables (Table 5). Significant predictors were in general the same parameters in both sexes except that QT_ea_ and the rate‐adjusted T_p_T_e_ interval ([T_p_T_e_]_a_) were significant predictors in men but not in women. HRs were particularly high for some of the ECG predictors in women: in 9 of them, HR was >2‐fold and in 7 of them >3‐fold. Five predictors in both men and women remained significant in the fully adjusted multivariable model.

**Table 5.** Hazard Ratios With 95% Confidence Intervals for Sudden Cardiac Death in Men and Women

	Men				Women			
	Univariable*	*P* Value	Multivariable*	*P* Value	Univariable	*P* Value	Multivariable	*P* Value
QRS duration, ms	1.15 (0.71 to 1.87)	0.564	1.11 (0.66 to 1.84)	0.701	1.53 (0.83 to 2.82)	0.173	1.08 (0.57 to 2.05)	0.804
RNDPV,* μV	1.18 (0.73 to 1.90)	0.503	1.09 (0.64 to 1.84)	0.759	1.25 (0.64 to 2.46)	0.511	0.84 (0.41 to 1.69)	0.617
QT_ea_,* ms	2.28 (1.50 to 3.48)	<0.001	1.94 (1.25 to 3.01)	0.003	1.63 (0.87 to 3.03)	0.126	1.18 (0.63 to 2.23)	0.605
T_p_T_xd_,* ms	1.24 (0.78 to 2.00)	0.354	1.11 (0.69 to 1.80)	0.663	1.79 (0.97 to 3.30)	0.061	1.34 (0.71 to 2.51)	0.366
θ(T_p_T_e_)_a_,* ms	1.63 (1.05 to 2.55)	0.031	1.44 (0.91 to 2.29)	0.120	1.69 (0.91 to 3.15)	0.010	0.90 (0.47 to 1.73)	0.748
RT_epi_,* ms	1.82 (1.17 to 2.83)	0.008	1.42 (0.89 to 2.26)	0.145	2.09 (1.15 to 3.82)	0.016	1.55 (0.83 to 2.90)	0.165
θ(R_m_|T_m_),* (°)	2.29 (1.51 to 3.48)	<0.001	1.54 (0.99 to 2.41)	0.058	4.91 (2.78 to 8.67)	<0.001	2.36 (1.27 to 4.39)	0.003
θ(T_p_|Tr_ef_),* (°)	2.91 (1.93 to 4.38)	<0.001	2.22 (1.43 to 3.43)	<0.001	5.90 (3.32 to 10.47)	<0.001	2.59 (1.39 to 4.82)	0.003
θ(T_init_|T_term_),* (°)	2.34*** (1.54 to 3.56)	<0.001	1.68 (1.07 to 2.62)	0.023	3.35 (1.89 to 5.93)	<0.001	1.47 (0.79 to 2.72)	0.226
T complexity*	1.20 (0.75 to 1.93)	0.451	0.66 (0.60 to 1.62)	0.958	1.88 (1.03 to 3.44)	0.039	1.31 (0.71 to 2.43)	0.388
TaVR, μV	2.52 (1.67 to 3.82)	<0.001	1.64 (1.04 to 2.58)	0.032	3.94 (2.24 to 6.95)	<0.001	1.79 (0.97 to 3.29)	0.063
ST_o_V,* μV	1.53 (0.97 to 2.40)	0.067	1.31 (0.78 to 2.19)		1.60 (0.85 to 3.02)	0.149	0.66 (0.34 to 1.32)	0.242
T_o_V, μV	1.18 (0.73 to 1.90)	0.500	1.16 (0.68 to 1.98)	0.592	1.02 (0.51 to 2.06	0.948	1.27 (0.62 to 2.62)	0.509
T_p_V, μV	1.17 (0.73 to 1.89)	0.512	1.41 (0.84 to 2.37)	0.192	1.80 (0.98 to 3.32)	0.223	0.78 (0.33 to 1.89)	0.585
T_o_V/T_p_V	1.62 (1.04 to 2.53)	0.034	1.18 (0.71 to 1.98)	0.5338	2.69 (1.51 to 4.79)	<0.001	0.93 (0.50 to 1.74)	0.818

HRs were evaluated for quintile 5 (quintile 1 for QT_oa_, T_amp_.aVL, and T_p_V) as the test group, with the remaining 4 quintiles as the reference group. HR indicates hazard ratio; CI, confidence interval; CHD, coronary heart disease; HDL, high‐density lipoprotein; LDL, low‐density lipoprotein.

* Univariable refers to unadjusted single ECG variable model and multivariable to single ECG variable multivariable‐adjusted risk model adjusted for age, race, education level, smoking status, alcohol status, asthma, cancer, diabetes, hypertension, family history of CHD and stroke, body mass index, systolic and diastolic blood pressure, HDL, LDL, triglycerides, white blood count, glucose, creatinine, and uric acid.

* RNDPV, QRS nondipolar voltage from singular value decomposition (square root of pooled variance of components 4 to 8).

* QT_ea_, rate‐adjusted QRe where QT_ea_=QT_e_+127 (1−RR) for men and QT_ea_=QT_e_+136 (1−RR) for women.

* T_p_T_xd_,T_p_T_xd_ interval representing dispersion of the initial left ventricular repolarization.

* θ(T_p_T_e_)a, global repolarization time dispersion (interval from QT_pa_ to QT_ea_).

* RT_epi_ denotes subepicardial repolarization time (see Methods and Results section).

* θ(R_m_|T_m_), spatial angle between mean QRS and T vectors.

* θ(T_p_|T_ref_), spatial angle between T_p_ vector and the T reference (T_ref_) vector with sex‐ and race‐specific unit vectors as follows: white men, 0.619; 0.483, −0.619; black men, 0.574; 0.390, −0.720; white women, 0.721; 0.583, −0.370; black women, 0.776; 0.504, −0.379).

* θ(T_init_|T_term_), spatial angle between the initial T vectors from quintiles 1 to 3 and the terminal T vectors from quintiles 4 to 5.

* T wave complexity, ratio of the second to the first principal component from singular value decomposition of the T wave.

* Symbol “V” in ST_o_V, T_o_V, and T_p_V refers to spatial magnitudes of the ST_o_, T_o_, and T_p_ vectors, respectively.

### Independent Predictors of CHD Death and SCD

Angular variables were commonly chosen as independent predictors of CHD death and SCD (Table 6). In addition, QT_ea_ was a significant independent predictor of CHD death and SCD in men, TV1 an independent predictor of CHD death and SCD in women, and TaVR an independent predictor of CHD death in men. Risk levels in women for both CHD death and SCD were stronger than in men. In terms of the magnitude of increased risk of CHD death for these ECG predictors, in men TaVR and QT_ea_ were the strongest predictors, with a 2‐fold increased risk for both variables. Also significant independent predictors were θ(R_m_|T_m_) and θ(T_init_|T_term_) with an >1.5‐fold increased risk (although the *P* values were marginally significant). In women, the risk of CHD death was increased 2‐fold for θ(R_m_|T_m_) and increasd 1.7‐fold for θ(T_p_|T_ref_).

**Table 6.** Hazard Ratios (95% Confidence Limits) for Independent Predictors* of Coronary Heart Disease and Sudden Cardiac Deaths by Sex

Men			Women		
	HR (95% CI)	*P* Value		HR (95% CI)	*P* Value
Coronary heart disease death					
θ(R_m_|T_m_),* (°)	1.62 (1.03, 2.55)	0.037	θ(R_m_|T_m_), (°)	2.04 (1.36, 3.07)	<0.001
θ(T_init_|T_term_),* (°)	1.50^∥^ (1.00, 2.25)	0.049	θ(T_p_|T_ref_),* (°)	1.70 (1.11, 2.61)	0.015
TaVR, μV	2.05 (1.20, 3.49)	0.009	TV1, μV	2.03 (1.40, 2.96)	<0.001
QT_ea_,* ms	2.05 (1.20, 3.49)	0.004			
Sudden cardiac death					
θ(T_p_|T_ref_), (°)	1.91 (1.14, 3.20)	0.013	θ(T_p_|T_ref_),* (°)	3.55 (1.85, 6.81)	<0.001
θ(T_init_|T_term_), (°)	1.61 (1.01, 2.56)	0.044	θ(R_m_|T_m_), (°)	2.28 (1.17, 4.45)	0.016
QT_ea_, ms	1.98 (1.29, 3.03)	0.002	TV1, μV	2.26 (1.20, 4.25)	0.012

* Independent predictors were obtained by entering the predictors that were significant in multivariable adjusted models in Tables 4 and 5 simultaneously into the Cox regression model and adjusting each variable to other significant predictors.

* θ(R_m_|T_m_), spatial angle between mean QRS and T vectors.

* θ(T_init_|T_term_), spatial angle between the initial T vectors from quintiles 1 to 3 and the terminal T vectors from quintiles 4 to 5.

* QT_ea_, rate‐adjusted QT_e_ where QT_ea_=QT_e_+127 (1−RR) for men and QT_ea_=QT_e_+136 (1−RR) for women.

* θ(T_p_|T_ref_), spatial angle between T_p_ and T_ref_ vectors.

Independent predictors of SCD in men were θ(T_p_|T_ref_) and QT_ea_, with a 2‐fold increased risk, and θ(T_init_|T_term_), with a 1.6‐fold increased risk. In women the strongest independent predictor of SCD was θ(T_p_|T_ref_) (HR, 3.55; CI, 1.85 to 6.81; *P*<0.001). In addition, the risk of SCD for θ(R_m_|T_m_) and TV1 was increased >2‐fold for both.

In terms of sex differences, the risk levels in women for both CHD death and SCD were stronger than in men. Another notable sex difference was that QT_ea_ was a significant independent predictor of CHD death and SCD in men but not in women.

## Discussion

A majority of the 18 ECG parameters were significant CHD death and SCD predictors when evaluated as unadjusted single ECG variables, and many remained significant in multivariable‐adjusted models. Notable among these predictors were θ(R_m_|T_m_), the spatial angle between the mean QRS and T vectors, and θ(T_p_|T_ref_), the spatial angle between Tpeak and the normal T reference vector. θ(R_m_|T_m_) is a measure of the overall deviation angle between depolarization and repolarization sequences, and θ(T_p_|T_ref_) is a measure of deviation of the direction of the repolarization sequence from the normal reference direction during regional cross‐mural repolarization of the left ventricular lateral wall. θ(R_m_|T_m_) was also an independent predictor for CHD death in men, with a 62% increased risk, and in women, with a 2‐fold increased risk, and θ(T_p_|T_ref_) was a strong independent predictor for SCD, with a nearly 2‐fold increased risk in men and a >3‐fold increased risk in women. It is worth noting that increased CHD death and SCD risk were observed in 20% of men and women (the upper quintile), with a relatively moderate widening (23° to 30°) of θ(T_p_|T_ref_) and θ(T_init_|T_term_). QT prolongation was an independent predictor for CHD death and SCD in men but not in women.

### Validity of the Repolarization Model

As noted in the Methods section, RT_p_ is computed as a function of the QT_pa_, which is the key parameter in the algorithms for RT_p_ and RT_xd_. RT_p_ is conceived by the repolarization model to represent the RT of LV myocytes, which repolarize at the time of T_p_ during the initial fast phase of LV lateral wall repolarization. It is noted from the algorithms for deriving RT_p_ as a function of QT_pa_ that the RT_p_ is modified by the cosine of θ(T_p_|T_ref_). These relationships imply that RT_p_=QT_pa_ if and only if θ(T_p_|T_ref_)=0° and that QT_pa_ is assigned to RT_xd_ if θ(T_p_|T_ref_)=180°; if θ(T_p_|T_ref_) is 90°, RT_p_ and RT_xd_ are both equal to QT_pa_. These functional relationships in the repolarization model are based on consideration of potential theory as applied to the generation of T wave, and they differ from the notions from some electrophysiological reports on animal models using wedge preparations that T_p_ timing always coincides with QT_epi_.^23–26^ Potential theory supports the assertion that at the time of RT_p_ the majority of LV lateral wall myocytes are in phase 3 of their action potential and that RT_p_ timing coincides with the timing of the global T_p._ It thus seems a rational proposition to maintain the labels RT_epi_ and RT_xd_ as derived by modifying RT_p_ by θ(T_p_|T_ref_). In a strict sense, the label RT_epi_ refers to the RT of subepicardial myocyte layers and should be considered as a representative value of LV epicardial RT and not RT at any specific epicardial location.

Potential theory is also compatible with the notion that at the time of T_p_ and RT_p_, LV lateral wall subepicardial myocytes in the region where repolarization starts earliest have already reached phase 4 of their action potential, no longer contribute to the generation of the T wave, and T amplitude starts to decline. This occurrence is the likely explanation for electrophysiological data relating the timing of QT_p_ with QT_epi_ in normal repolarization.^23–26^ Parameter T_xd_ in the repolarization model is the inflexion point (the minimum slope) at the global T‐wave downstroke, considered by the repolarization model to occur when the largest number of LV myocytes leaves phase 3 of their action potential within the same increment of RT. With the normal direction of the RT sequence, this conceivably occurs when the majority of subendocardial myocytes reach their resting potential. Spatial direction of repolarization during the T_p_T_xd_ interval is diametrically opposite to the direction of the T_p_ vector, and T_p_T_xd_ is the magnitude of the temporal RT gradient vector representing RT dispersion during the T_p_T_xd_ interval dominated by the LV lateral wall repolarization. Contrary to the notions from electrophysiological studies suggesting that LV lateral wall repolarization is perpendicular to the epicardial surface,^23–25^ there is consistent evidence that the spatial LV repolarization sequence remains throughout repolarization closely in the direction from inferior‐left‐anterior to superior‐right‐posterior, approximately in the direction of the lead vector of aVR but with a posterior component.^21–22^ This implies that LV lateral wall repolarization is cross‐mural, oblique rather than perpendicular to the epicardial surface.^10,21–22,27^

### Mechanisms of Generation of Repolarization Abnormalities as Independent Predictors of CHD Death and SCD

Anterior‐right rotation of the T_p_ vector is a predominant determinant of widened θ(R_m_|T_m_) and θ(T_p_|T_ref_) angles.^10,21^ T_p_ vector rotation closer to the aVR lead axis results in decreased (less negative) TaVR amplitude that ultimately becomes positive with a more pronounced widening of θ(T_p_|T_ref_). Altered direction of the repolarization sequence may reflect subepicardial action potential duration shortening such as takes place in anterior subepicardial myocardial ischemia.^21^ Thus, the increased θ(T_p_|T_ref_) observed in the present study as a common predictor for CHD death and SCD may possibly be an early marker of evolving subclinical CHD in men and women free from manifest CVD. θ(T_p_|T_ref_) widening also reflects a gradual change from the normal predominantly reverse sequence of the cross‐mural left ventricular wall repolarization to a concordant repolarization with respect to depolarization and increasing dyssynchrony of repolarization^10^ that in turn has been postulated to be associated with increased dyssynchrony of ventricular repolarization as another possible risk mechanism.^26^

**Figure 2.**
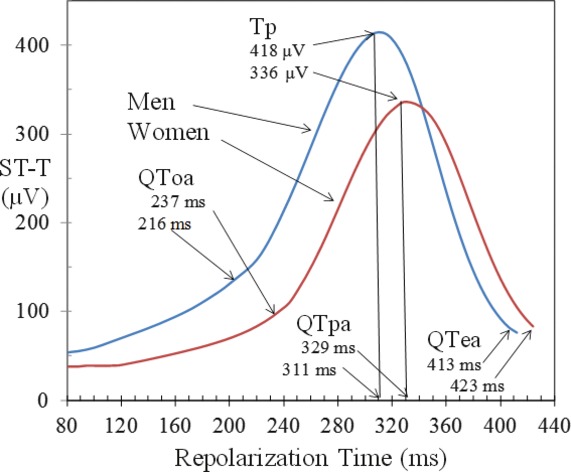
Mean global ST‐T waveforms in men and women free from cardiovascular disease highlighting sex differences in rate‐adjusted QTpeak (QT_pa_), QTend (QT_ea_), and QTonset (QT_oa_) intervals and Tpeak (T_p_) amplitudes. The ST‐T curve was generated by sampling the ST‐T vector magnitude function between the end of QRS and the end of the T wave (T_e_) at 60 equally spaced sample points, subsequently aligning the Tpeak (T_p_) time with the mean QT_pa_ and rescaling the temporal RT axis back to the original to match T_p_T_e_ and T_o_T_p_ intervals with the mean QT_pa_ and T_oa_–T_pa_ intervals of men and women. Compared with that in women, repolarization in men starts earlier, the ST segment has a steeper upslope, and T_p_ is shifted to the left, corresponding to an earlier end of epicardial repolarization as reflected by an 18‐ms shorter QT_pa_ in men. CVD indicates cardiovascular disease.

QT_ea_ was not an independent predictor for either end point in women, but in men it was a significant independent predictor, with a 48% increased risk for CHD death and a 98% increased risk for SCD. Sex difference in QT is actually not a result of QT prolongation in women, as commonly claimed, but arises from pronounced QT shortening in adolescent boys.^28^ QT gradually prolongs with age in adult men, and the sex difference becomes small or vanishes after middle age. Although QT_ea_ is a measure of the global RT, QT_pa_ and RT_epi_ are measures of regional RT. The present investigation revealed an 18‐ms sex difference in QT_pa_ (Figure 2), indicating that the sex difference in RT_p_ remains more pronounced in middle‐aged men and women than the 10‐ms sex difference in QT_ea_ as listed in Table 3. It is not known whether prolonged regional repolarization time (RT_p_) might play some role in explaining the higher vulnerability of women than men to the proarrhythmic effects of cardioactive drugs.^29^

### Comparison With Previous Studies

Two recent publications in general population samples of men and women^30–31^ and 1 in men^32^ found QRS duration to be predictive of SCD. In our study population of CVD‐free men and women, QRS duration was a significant predictor only in the unadjusted single ECG variable risk model in women for CHD death.

Several publications have documented an increased mortality risk for a wide mean QRS|T angle^8–12^ and abnormal T‐wave axis.^33–34^ Various angular measures of altered repolarization sequence were the most common predictors for CHD death and SCD in our study. Increased T‐wave amplitude in aVR was reported to be a significant predictor of cardiac mortality in the general population of men and women^35^ and in a large clinical male population.^36^

The previous investigations cited above have evaluated ECG predictors of CHD death and SCD as single variables or as a limited group of variables. The present investigation is the first large‐scale population study with simultaneous evaluation of a comprehensive set of repolarization‐related parameters.

### Limitations of This Investigation

Although the multivariable models employed were adjusted for a variety of demographic and clinical factors, competing risk analysis was not performed. The primary objective of the study was to identify in CVD‐free men and women a subset of ECG parameters for future risk evaluation studies as potentially more sensitive predictors of CHD death and SCD than the QT interval.

### Clinical Significance and Avenues for Future Research

θ(R_m_|T_m_) and θ(T_p_|T_ref_), reflecting different aspects of ventricular repolarization, were found to be independent predictors of CHD death and SCD, and TaVR and TV1, readily available in standard ECG reports, were also independent predictors. Among notable sex differences, the risk levels for independent predictors for both CHD death and SCD were stronger in women than in men, and QT_ea_ was a significant predictor in men but not in women. These ECG variables identified as independent predictors of CHD death and SCD are the primary candidates that warrant consideration in risk evaluation studies. However, all the repolarization‐related parameters that were significant when evaluated as single variables need attention in the evaluation of possible markers of toxic drug effects using well‐validated annotated data files from drug trials.
